# Nicotinamide N-Methyltransferase (NNMT) and Liver Cancer: From Metabolic Networks to Therapeutic Targets

**DOI:** 10.3390/biom15050719

**Published:** 2025-05-14

**Authors:** Shi-Yan Lai, Xiao-Juan Zhu, Wei-Dong Sun, Shuang-Zhou Bi, Chen-Ying Zhang, An Liu, Jiang-Hua Li

**Affiliations:** Physical Education College, Jiangxi Normal University, Nanchang 330022, China; laishiyan@jxnu.edu.cn (S.-Y.L.); zhxjhh@jxnu.edu.cn (X.-J.Z.); sunweidong0@jxnu.edu.cn (W.-D.S.); bishuangzhou@jxnu.edu.cn (S.-Z.B.); 18738910347@jxnu.edu.cn (C.-Y.Z.); liuan@jxnu.edu.cn (A.L.)

**Keywords:** NNMT, liver cancer, NAD^+^ metabolism, Hcy level, prognosis, therapeutic targets

## Abstract

Hepatocellular carcinoma (HCC), the predominant form of primary liver cancer, remains a global health challenge with limited therapeutic options and high mortality rates. Despite advances in understanding its molecular pathogenesis, the role of metabolic reprogramming in HCC progression and therapy resistance demands further exploration. Nicotinamide N-methyltransferase (NNMT), a metabolic enzyme central to NAD^+^ and methionine cycles, has emerged as a critical regulator of tumorigenesis across cancers. However, its tissue-specific mechanisms in HCC—particularly in the context of viral hepatitis and methionine cycle dependency—remain understudied. This review systematically synthesizes current evidence on NNMT’s dual role in HCC: (1) driving NAD^+^ depletion and homocysteine (Hcy) accumulation via metabolic dysregulation, (2) promoting malignant phenotypes (proliferation, invasion, metastasis, and drug resistance), and (3) serving as a prognostic biomarker and therapeutic target. We highlight how NNMT intersects with epigenetic modifications, immune evasion, and metabolic vulnerabilities unique to HCC. Additionally, we critically evaluate NNMT inhibitors, RNA-based therapies, and non-pharmacological strategies (e.g., exercise) as novel interventions. By bridging gaps between NNMT’s molecular mechanisms and clinical relevance, this review provides a roadmap for advancing NNMT-targeted therapies and underscores the urgency of addressing challenges in biomarker validation, inhibitor specificity, and translational efficacy. Our work positions NNMT not only as a metabolic linchpin in HCC but also as a promising candidate for precision oncology.

## 1. Introduction

Primary liver cancer (PLC) ranks as the sixth most prevalent type of cancer globally and the third major contributor to cancer-related mortality [1]. PLC primarily consists of three types: intrahepatic cholangiocarcinoma (ICC), hepatocellular carcinoma (HCC), and combined hepatocellular-cholangiocarcinoma (cHCC-CCA). HCC constitutes approximately 90% of primary liver cancer cases [2], exhibiting both high incidence and mortality rates [3]. Growing evidence suggests that early stages of HCC development and its precursor lesions share common features regulated by genetic and epigenetic mechanisms [4,5]. Additionally, alterations in various methylation processes have been observed in liver diseases, including an increase in nicotinamide methylation in cirrhosis patients [6,7,8,9]. Therefore, gaining a comprehensive insight into the etiology of liver cancer and pinpointing viable therapeutic targets are crucial steps in improving the survival rates and overall well-being of patients suffering from this disease.

Emerging evidence highlights the dual role of Nicotinamide N-methyltransferase (NNMT) across solid tumors, with both conserved and tissue-specific mechanisms. In breast cancer, NNMT stabilizes SIRT1 to drive chemoresistance [10], while, in pancreatic cancer, it paradoxically suppresses tumorigenesis by altering oncometabolite profiles [11]. Notably, NNMT’s impact on NAD^+^ depletion and methionine cycle dysregulation is a shared hallmark in cancers such as HCC and ovarian tumors [12]; yet, its functional outcomes diverge depending on cellular context. For instance, NNMT promotes immune evasion in HCC through H3K27me3-mediated CD44 upregulation, whereas, in melanoma, it primarily induces chemoresistance via epigenetic silencing [13]. These comparisons underscore the need to dissect NNMT’s role through both pan-cancer and tissue-specific lenses, particularly in liver cancer where viral hepatitis and unique methionine cycle dependencies amplify its pro-tumorigenic effects.

Elevated NNMT levels have been observed in various liver cancers, and this upregulation is linked to cancer growth and progression [14,15,16]. NNMT, a key metabolic enzyme, regulates the levels of S-adenosyl-L-homocysteine (SAH), N^1^-methylnicotinamide (MNAM), S-adenosyl-L-methionine (SAM), and nicotinamide (NAM) in liver cancer cells. It plays a crucial role in NAD^+^-mediated signaling pathways and homocysteine (Hcy) metabolism by utilizing NAM, a precursor to NAD^+^, and producing SAH, which is a precursor to Hcy [17,18]. NNMT dysregulation is implicated in multiple pathological conditions. In breast cancer, a high NNMT expression correlates with poor survival and adverse chemotherapy responses [10]. In pancreatic cancer, it modulates the metabolome to suppress malignant behaviors and tumorigenesis [11]. NNMT inhibition improves metabolic parameters, including energy expenditure, fat accumulation, insulin sensitivity, glucose tolerance, and fasting glucose [19]. In neurodegenerative diseases, NNMT dysregulation drives pathogenesis via NAD^+^ depletion and metabolic disruption [20]. Although the specific mechanisms by which NNMT influences liver cancer progression and its potential as a therapeutic target remain poorly understood, accumulating evidence suggests that NNMT plays significant roles in diagnosis, treatment, and prognosis. This review delves into the regulatory mechanisms governing NNMT expression in liver cancer and its association with malignant behaviors.

## 2. Metabolic Pathways Linking NNMT to Liver Cancer

**Figure 1:**
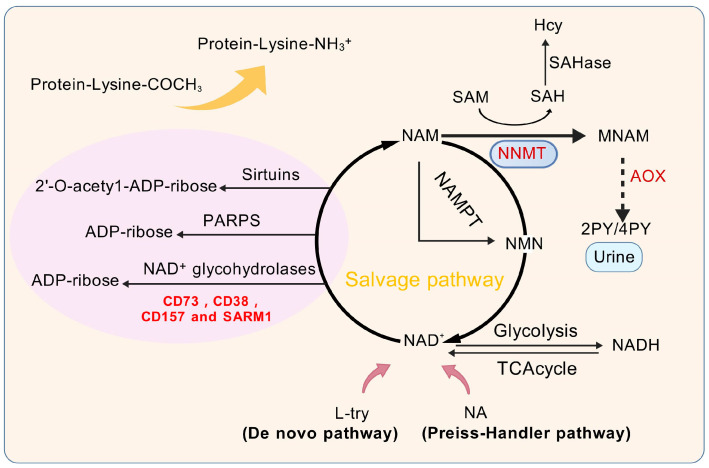
Physiological functions of NNMT and its metabolic pathways. L-try, L-tryptophan; NA, Nicotinic acid; NAD^+^, Oxidized form of nicotinamide adenine dinucleotide; 4 py, 4-Pyridinone; 2 py, 2-Pyridinone; AOX, Aldehyde oxidase; NAM, Nicotinamide; SAHase, S-adenosyl-L-homocysteine hydrolase; SAM, S-adenosyl-methionine; SAH, S-adenosyl-L-homocysteine; Hcy, Homocysteine; NNMT, Nicotinamide n-methyltransferase; MNAM, N^1^-Methylnicotinamide; NAD^+^ glycohydrolases, Enzymes like CD73; CD38; CD157 and SARM1, Sterile Alpha and TIR motif-containing protein 1; NAMPT, Nicotinamide Phosphoribosyltransferase; TCA cycle, Tricarboxylic Acid Cycle; Sirtuins, A family of NAD^+^-dependent proteins; PARPs, Poly(ADP-ribose) polymerases; NADH, Reduced form of nicotinamide adenine dinucleotide.

As depicted in Figure 1, NNMT catalyzes the methylation of NAM by transferring a methyl group from SAM, leading to the production of MNAM and SAH. As a pivotal methyl donor, SAM initiates a multitude of methylation reactions across the nuclear and cytoplasmic compartments. Notably, somatic mutations in the substrate-binding domain (e.g., R17C) have been shown to reduce NNMT methyltransferase activity by 40–60% [21], directly impairing SAM-to-SAH conversion efficiency. This mutational disruption creates a self-reinforcing loop: a diminished enzymatic capacity exacerbates SAM accumulation while reducing SAH-mediated feedback inhibition, ultimately driving DNA hypomethylation [22]. The modulation of NAM, SAH, SAM, and MNAM by NNMT undergoes fluctuating alterations, which are contingent upon the activities of other enzymes within distinct cellular contexts. Recent research suggests that the generation of MNAM is solely reliant on the action of NNMT, whereas the formation and utilization of SAM and NAM are influenced by the collaborative effects of NNMT and an array of other enzymes. Under normal conditions in liver tissue, the equilibrium concentrations of SAH, NAD^+^, SAM, and NAM not significantly influenced by the activity of NNMT. Conversely, in individuals with liver cancer, the diminished functionality of glycine N-methyltransferase (GNMT) increases the dependency on NNMT for SAM synthesis, given that GNMT is the predominant enzyme accountable for the consumption of SAM [23]. This dependency is amplified in tumors harboring NNMT mutations. TCGA-LIHC data reveal that 4.2% of HCC cases exhibit NNMT genetic alterations, predominantly deep deletions and missense mutations [21]. Structural modeling indicates these mutations destabilize the enzyme’s catalytic pocket, reducing the SAM binding affinity by 3.8-fold. In this pathological state, the disruption of the methyl donor balance, along with the dysregulation of NNMT, SAM, SAH, MNAM, and NAM, may lead to abnormalities in the regulation of NNMT’s metabolic pathways, potentially linking these changes to the pathogenesis of liver cancer. The metabolic function of NNMT is believed to diminish the levels of NAM, an essential precursor for the production of NAD^+^, and, concurrently, leads to the formation of SAH, a pivotal compound in the pathway for Hcy synthesis, as illustrated in Figure 1. Mutant NNMT proteins exacerbate this metabolic crisis: R17C variants show a 62% reduced NAD^+^ salvage capacity compared to the wild-type ones (*p* = 0.003) while increasing Hcy production by 1.9-fold [21]. As a result, NNMT can downregulate NAD^+^ levels and upregulate Hcy levels. A significant body of research indicates that the depletion of NAD^+^ levels and the concurrent rise in Hcy play pivotal roles in the progression of hepatocellular carcinoma [19,24]. This subsequent section will delve into the metabolic regulatory functions of NNMT within the context of liver cancer.

### 2.1. NAD^+^ Cycle

NAD^+^ is an essential coenzyme integral to cellular energy metabolism, playing key roles in maintaining cellular homeostasis, facilitating DNA repair, mediating redox reactions, and regulating epigenetic signaling pathways. NAD^+^, nicotinamide adenine dinucleotide in its oxidized state, is crucial for a variety of enzymatic reactions critical to energy metabolism, including glycolysis, oxidative phosphorylation, the citric acid cycle, and the beta-oxidation of fatty acids [25]. Indeed, the NAD^+^ content can also be modulated by the extracellular conversion of nicotinamide mononucleotide (NMN) and nicotinamide riboside (NR) by CD73 [26]. Reduced levels of NAD^+^ are seen as a key factor in the NNMT-driven progression of liver cancer. As depicted in Figure 1, NAD^+^ biosynthesis occurs via three principal routes: the salvage pathway, the Preiss–Handler mechanism, and the de novo synthesis. The salvage pathway is the predominant route, contributing over 85% of total NAD^+^ synthesis [27,28,29,30]. In contrast to the Preiss–Handler mechanism and the de novo synthesis, which depend on substrates like nicotinic acid (NA) and tryptophan (L-try), the salvage pathway is pivotal in the biosynthesis of NAD^+^, predominantly using NAM as the core substrate [31]. Nonetheless, NNMT facilitates the methylation reaction of NAM, yielding MNAM as a product. This compound is either excreted through urine or metabolized further by aldehyde oxidase (AOX), resulting in the formation of 4-pyridone (4 py) and 2-pyridone (2 py). Irrespective of the mode of excretion, NNMT’s conversion of NAM into MNAM impedes NAM’s recycling in the NAD^+^ salvage pathway, directly affecting NAD^+^ production and leading to its depletion. NAD^+^ plays a regulatory role in a multitude of pivotal enzymes, encompassing NAD^+^-glycohydrolases like CD38 and CD157, sirtuins, and poly(ADP-ribose) polymerases (PARPs). These enzymes are integral to the dynamics of cancer progression, as depicted in Figure 1. Additionally, NAD^+^ is essential for the deacetylase activity of PARP proteins in DNA damage and repair, as well as for ADP-ribosylation reactions. Certain NAD^+^ hydrolases reduce NAD^+^ levels by transforming it into cyclic ADP-ribose or ADP-ribose. For example, CD38, in particular, has a profound effect on the functioning of the immune system. Given that NAD^+^ is not readily available from dietary intake, the significance of its biosynthetic pathways cannot be overstated. This underscores the significance of NAD^+^ in various pathological and physiological processes, highlighting the critical role of NAD^+^ regulation in liver cancer research and overall human health [32].

#### 2.1.1. Association of Reduced NAD^+^ Levels with Liver Cancer

NAD^+^ serves as an essential cofactor in the metabolic process of transforming carbohydrates into lipids and the oxidation of energy substrates [33], occupying a pivotal position in metabolic pathways. A reduction in NAD^+^ levels could represent a key underlying mechanism through which NNMT facilitates the progression of liver cancer, substantially elevating the risk of oncogenesis.

Abundant studies have demonstrated that genetically deleting NNMT leads to a substantial elevation of intracellular NAD^+^ concentrations within adipose cells in mice [34]. Jin et al. [35] demonstrated that the NAD^+^-dependent deacetylase SIRT3 is capable of removing acetyl groups from lysine residues. It is expressed at low levels in liver cancer and is considered a tumor suppressor in liver cancer. Consistent with these findings, the targeted deletion of NNMT in cancer-associated fibroblasts (CAFs) has been observed to boost NAD^+^ concentrations [36]. Preserving the homeostasis of intracellular NAD^+^ is essential, as it is regulated by the balance of NAD^+^ to NADH, which can theoretically be interconverted without being consumed [37]. Consequently, the intracellular amounts of NAD^+^ and NADH should ideally remain balanced. However, in reality, the concentration of NAD^+^ is 600 to 1100 times higher than that of NADH [38], a significant disparity due to NAD^+^’s involvement in numerous non-redox biological pathways, encompassing DNA repair, cellular communication, protein post-translational modifications, mitochondrial function control, inflammation response, aging, and apoptosis [25]. In these non-redox mechanisms, NAD^+^ serves as a key substrate for various enzymes, including sirtuins, PARPs, and cADPR synthetase, the latter being part of the ADP-ribosyl cyclase family. Such enzymes utilize NAD^+^ as a donor of ADP-ribose, simultaneously producing NAM as a byproduct throughout these metabolic processes. Chen et al. [39] showed that inhibiting 6-phosphogluconate dehydrogenase (6 PGD) triggers the activation of AMP-activated protein kinase (AMPK) and acetyl-CoA carboxylase 1 (ACC1). This activation results in a decrease in the NADPH/NAD^+^ and NADH ratios within HCC, consequently diminishing SIRT1 function and inducing oxidative stress. In contrast, the absence of AMPK substantially reversed the impact of physcion, a selective 6 PGD inhibitor, on lowering the NADPH/NAD^+^ ratio and curbing proliferation and viability, thereby validating AMPK’s role as an upstream activator in the inhibition of 6 PGD within HCC cells. Elevated levels of NNMT expression deplete NAD^+^ reserves and interfere with the normal regulation of glucose metabolism [40]. Glucose metabolism provides vital carbon sources and energy for the body, with the majority of human energy needs being derived from glucose. Intermediate metabolites of glucose metabolism, such as 6-phosphoglucose, dihydroxyacetone phosphate, pyruvate, acetyl-CoA, and oxaloacetate, act as carbon sources for the production of amino acids, nucleotides, fatty acids, and steroids, all of which are crucial for DNA demethylation processes. Thus, the decline in NAD^+^ levels constitutes a key mechanism through which NNMT impacts liver cancer cell proliferation and properties, consequently affecting the viability of healthy hepatic cells.

#### 2.1.2. Possible Mechanisms Linking NAD^+^ Cycle to Liver Cancer

**Figure 2:**
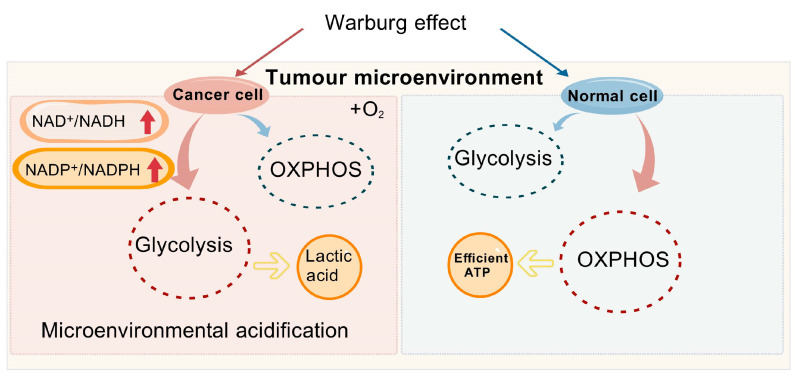
Figure 2 illustrates the potential mechanisms underlying the Warburg effect in cancer cells. NAD^+^, Oxidized form of nicotinamide adenine dinucleotide; NADH, Reduced form of nicotinamide adenine dinucleotide; NADP^+^, Nicotinamide Adenine Dinucleotide Phosphate; NADPH, Nicotinamide Adenine Dinucleotide Phosphate; OXPHOS, Oxidative Phosphorylation; ATP, Adenosine Triphosphate.

It is well-established that cancer cells modify their metabolic processes to support rapid cell division and survive in unfavorable conditions such as hypoxia and nutrient scarcity [41]. Cancer cells, in contrast to their normal counterparts, display elevated ratios of oxidized nicotinamide adenine dinucleotide phosphate (NADP^+^) to NADPH, as well as NAD^+^ to NADH, emphasizing the pivotal role of NAD^+^ in the metabolic shift that defines cancer metabolism [42]. Tumor cells often adapt to stressful environments and achieve unlimited proliferation through metabolic reprogramming, a characteristic that holds potential for non-invasive cancer diagnosis [43,44]. There are significant differences in energy acquisition between tumor cells and normal cells. While normal cells predominantly rely on oxidative phosphorylation (OXPHOS) for energy production, cancer cells often prefer glycolysis as their primary energy source, even when ample oxygen is available, a metabolic adaptation referred to as the “Warburg effect” (Figure 2) [45].

The Warburg effect [46] delineates the preferred metabolic route of cancer cells, favoring glycolysis over OXPHOS, even in the presence of abundant oxygen. Cancer cells predominantly utilize glucose for energy production and are reliant on NAD^+^ to catalyze essential metabolic processes [25]. Additionally, several mechanisms promote the development of liver cancer, including genetic mutations, pathway activation, and somatic DNA alterations in liver cancer cells, such as mutations in the TP53 [47], TERT promoter [48], and genes involved in WNT signaling [49]. These changes affect critical cellular functions such as the control of cell division, differentiation, and apoptosis. Immune evasion also plays a central role in liver cancer progression. Metabolic reprogramming within the tumor microenvironment (TME) meets the energy demands of both cancer cells and the immune components of the tumor, thereby driving disease progression. Tumor cells promote rapid proliferation by upregulating glycolysis, gluconeogenesis, and β-oxidation, resulting in a glucose-deficient but lactate-rich environment that alters immune responses in the liver, enabling immune evasion by tumor cells [50]. Consequently, neoplastic cells markedly enhance their glucose intake to support their unchecked growth and proliferation [51,52]. For the maintenance of elevated glycolytic rates, it is crucial that we increase NAD^+^ levels, considering its critical function in key metabolic processes. For instance, glyceraldehyde-3-phosphate dehydrogenase (GAPDH) catalyzes the transformation of glyceraldehyde-3-phosphate into lactate. Although OXPHOS generates ATP more efficiently (producing 36 ATP molecules per mole of glucose), cancer cells tend to favor glycolysis due to their higher ATP demand, producing only two ATP molecules per mole of glucose [53]. The swift generation of energy grants hepatic cancer cells a competitive edge in environments where nutrients are scarce, enabling them to proliferate more rapidly than the surrounding stromal cells. Furthermore, the lactate generated through glycolysis leads to the acidification of the tumor microenvironment, a condition that fosters the invasiveness of cancer cells and dampens immune responses against the tumor.

NAM, acting as a precursor for NAD^+^, potently inhibits sirtuin enzymes. NNMT regulates the synthesis of NAD^+^ through the metabolic conversion of NAM. Empirical evidence indicates that NNMT has the ability to enhance the expression and activation of sirtuins [54]. Pioneering research by Estep et al. [55] initially revealed the link between NNMT and the sirtuin family, influencing both their expression and functionality. They observed increased levels of NNMT and Sirt1 expression following caloric restriction in mice. Further studies [56] have indicated that the activation of Sirt1 promotes gluconeogenesis, inhibits cholesterol synthesis, and suppresses lipogenesis, implying that NNMT could be pivotal in modulating these metabolic pathways through the stabilization of the Sirt1 protein [57]. B et al. [58] demonstrated that SIRT1 and SIRT3 are essential for enhancing mitochondrial performance under conditions of glucose scarcity. This enhancement is mediated through the activation of the AMPK-p53-PGC1α signaling axis in HCC cells. This highlights the metabolic role of SIRT1 in HCC pathogenesis and suggests its potential relevance for future liver cancer treatments. Conversely, the addition of NAD^+^ precursors, including NA or NAM, may counteract these metabolic alterations, facilitating the re-establishment of cellular homeostasis. Additionally, Zhang et al. [59] investigated SIRT6 levels across a range of HCC cell lines, delving into its influence on cellular expansion and programmed cell death. By transfecting Huh-7 cells with plasmids for SIRT6 overexpression and small interfering RNA (siRNA) for silencing SIRT6, they found that SIRT6 overexpression increased Huh-7 cell proliferation. This effect was accompanied by the upregulation of Bcl-2, an anti-apoptotic protein, and the enhanced phosphorylation of the extracellular signal-regulated kinase (ERK). At the same time, SIRT6 overexpression reduced the levels of cleaved caspase-3, a key enzyme in the apoptosis execution phase, and Bax, a key pro-apoptotic protein from the Bcl-2 protein family. The results suggest that SIRT6 regulates ERK1/2 signaling to influence HCC cell proliferation and apoptosis, thereby altering the activation state of endogenous apoptosis pathways. Although SIRT6 is considered a promising target for HCC therapy, its exact mechanisms of action still require further investigation.

PARP is a DNA repair enzyme predominantly located in the nucleus of various cell types. This enzyme catalyzes the conjugation of ADP-ribose from the cofactor NAD^+^ to specific protein targets. NAD^+^ is the sole substrate for PARPs, and the reaction produces poly(ADP-ribose) (PAR) chains along with NAM [54]. PARP1, the most studied and prominent member, is instrumental in the repair of single-strand DNA breaks (SSBs) and is commonly hailed as the custodian of DNA integrity. When DNA is damaged, PARP1 is activated, and, in the presence of genotoxic stress, the excessive activation of PARP1 leads to a significant depletion of ATP and NAD^+^. This depletion negatively impacts the activity of sirtuins and cellular stability, potentially leading to cell death [60]. Beyond its role in DNA repair, PARP1 is implicated in a multitude of cellular activities, such as the promotion of cell growth, modulation of cell cycle progression, control of gene expression, mediation of inflammatory responses, and orchestration of cellular destiny. The cellular activation of PARP1 can be triggered by DNA damage or elevated NAD^+^ levels. The enzyme’s function as a promoter or suppressor of tumorigenesis is contingent upon the degree of its activation. In instances of extensive DNA damage, PARP1 generally facilitates apoptosis by initiating p53-mediated pathways [61]. Notably, knockdown of the PARP1 results in elevated NAD^+^ levels, while knockdown of the PARP2 enhances SIRT1 expression through binding to and inhibiting the SIRT1 promoter [62]. In cancer cells, the excessive activation of PARPs significantly increases the catabolism of NAD^+^, resulting in a concomitant rise in NAM levels and a reduction in NAD^+^ concentrations, which may markedly reduce the deacetylase activity of SIRT1 [63]. During DNA repair, ADP-ribose units are polymerized at DNA breaks into nucleoproteins, including histones and transcription factors. Maintaining an appropriate level of NAM is crucial for preventing PARP degradation, thereby supporting the DNA repair process [64]. NNMT is crucial for the efficient conversion of NAM back into NAD^+^ within the recycling process. Research has underscored the pivotal function of PARP in sustaining NAD^+^ homeostasis. For example, Yang et al. [65] found that PARP1 is indeed highly expressed in human embryonic stem cells but gradually decreases as the cells differentiate into specified liver cells. In a xenograft mouse model, PARP1 expression was reactivated in residual HCC tumors following treatment with Sorafenib [66], which implies that PARP1 could have a substantial impact on stem cell potency and the development of resistance to Sorafenib in HCC. Sorafenib treatment induces highly active DNA damage repair signaling, which is essential for maintaining the pluripotency of stem cell in residual HCC tumors. Additionally, the PARP inhibitor Olaparib broadly suppresses DNA damage repair signaling and may enhance the overall remodeling of the pluripotency transcriptome, thereby improving the efficacy of Sorafenib in eliminating residual HCC tumors. PARP inhibition increases NAD^+^ levels, enhances SIRT1 activity, boosts mitochondrial function, and modulates liver cancer [67,68]. Overall, NNMT, by regulating the biosynthesis and metabolism of NAD^+^, profoundly influences PARP activity. The activation state of PARP is pivotal in regulating cell fate and is intimately connected to liver cancer treatment strategies and NAD^+^ metabolic balance.

**Figure 3:**
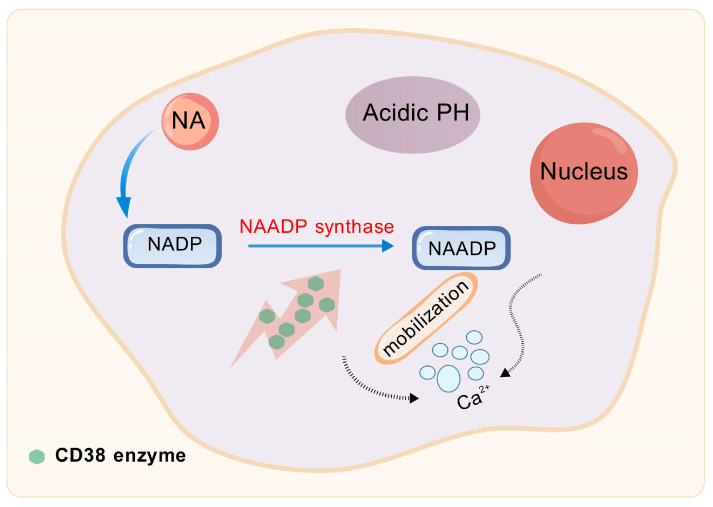
CD38 enzyme is involved in base exchange reactions. NA, Nicotinic acid; NADP, Nicotinamide Adenine Dinucleotide Phosphate; NAADP, Nicotinic Acid Adenine Dinucleotide Phosphate.

NAD^+^ glycohydrolases are a group of enzymes with specific catalytic functions, primarily including members such as CD73, CD38, CD157, and SARM1 (Sterile Alpha and TIR motif-containing protein 1). These enzymes primarily catalyze the hydrolysis of NAD^+^, severing the glycosidic bond within its molecular framework to yield NAM and ADP-ribose [69]. By regulating NAD^+^ levels, NAD^+^ glycohydrolases significantly impact the concentration of NAM within cells. Among these enzymes, CD38 stands out as the predominant NAD^+^ glycohydrolase, or NADase, in mammals and is extensively acknowledged for its pivotal role in regulating NAD^+^ metabolism [70,71]. CD38 also participates in base exchange reactions, and, under acidic pH conditions, it catalyzes the conversion of NADP to NAADP (nicotinic acid adenine dinucleotide phosphate) through its NAADP synthase activity, in the presence of NA [72]. Substances such as NAAD(P), cyclic ADP-ribose, and ADP-ribose function as significant second messengers for Ca^2+^ mobilization, highlighting CD38’s pivotal role in initiating Ca^2+^ signaling and influencing various diverse cellular activities, encompassing immune responses and cancer progression (as shown in Figure 3) [73]. For instance, by using CD38 siRNA-loaded extracellular vesicles (EVs/siCD38), the enzymatic activity of CD38 can be effectively inhibited, which subsequently reduces adenosine production. The inhibition leads to the dampening of liver cancer proliferation and the prevention of their metastatic spread in vitro studies [74]. Therefore, by modulating NAD^+^ availability, CD38 plays a crucial role in indirectly influencing DNA repair processes.

CD157, an essential component of the ADP-ribosyl cyclase family, exerts a significant influence on NAD^+^ metabolism [75]. Although direct studies linking CD157 enzyme activity to liver cancer are still limited, its potential mechanism can be explored from the perspective of NAD^+^ metabolism. Aberrations in NAD^+^ metabolism are recognized as major contributors to the onset and progression of liver cancer [76]. For instance, during the oncogene-induced development of HCC, the de novo synthesis of NAD^+^ is inhibited, leading to impaired DNA repair mechanisms, which, in turn, becomes a key factor in the advancement of cancer. As one of the critical enzymes in NAD^+^ metabolism, any dysregulation in the expression or activity of CD157 could potentially impact the development of liver cancer indirectly.

SARM1 is an enzyme with NAD^+^ hydrolase activity, and its activation can lead to axonal degeneration. Research by Figley et al. [77] has demonstrated a correlation between SARM1 activation and an increase in the ratio of NMN to NAD^+^. However, the regulatory effect of NNMT on SARM1 under physiological conditions, as well as the extent of this regulation, remains unclear. SARM1 facilitates the catabolism of NAD^+^ to produce NAM, and NNMT potentially modulates SARM1 function indirectly through the regulation of NAD^+^ synthesis. It has been shown that the NAD^+^ breakdown activity of SARM1 plays a crucial role in axonal degeneration [78]. While the direct link between SARM1 and liver cancer is not yet fully established, its involvement in neurodegenerative diseases—particularly its role as an NAD^+^ glycosidase in axonal degeneration—has garnered significant attention.

Overall, NAD^+^ production can be influenced by regulating the consumption of NAM. NAD^+^ is essential for modulating the activity of NAD^+^-dependent enzymes, including CD157 and CD38, sirtuins, and PARPs. Komatsu et al. [18] showed that the excessive activation of NNMT results in decreased NAD^+^ levels in the liver. This subsequently impairs the function of NAD^+^-dependent deacetylase Sirt3, resulting in a decreased activity of genes related to fatty acid oxidation. The interactions of these enzymes with liver cancer are closely linked, suggesting that heightened NNMT activity could potentially influence the regulatory mechanisms of these enzymes in an indirect manner.

### 2.2. Methionine Cycle

**Figure 4:**
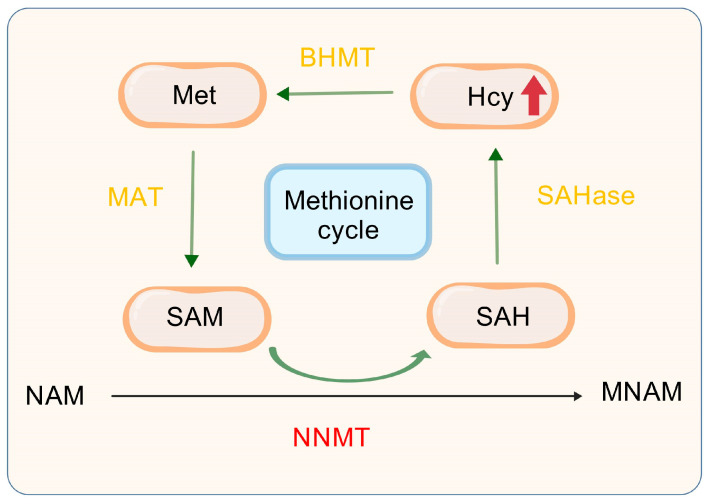
NNMT with methionine cycle. NAM, Nicotinamide; SAHase, S-adenosyl-L-homocysteine hydrolase; SAM, S-adenosyl-methionine; SAH, S-adenosyl-l-homocysteine; Hcy, Homocysteine; S-adenosyl-L-homocysteine; NNMT, Nicotinamide n-methyltransferase; MNAM, N^1^-Methylnicotinamide; Met, Methionine; BHMT, Betaine-Homocysteine Methyltransferase; MAT, Methionine Adenosyltransferase.

NNMT is a pivotal player in the methionine cycle, a key metabolic pathway in the body that involves the metabolism, regeneration, and methyl transfer reactions of methionine. These processes significantly impact the onset and progression of liver cancer. Figure 4 illustrates that NNMT facilitates the methylation of NAM, leading to the production of MNAM and SAH, with SAM serving as the methyl donor [79]. Subsequently, SAH undergoes hydrolysis catalyzed by SAHase, which represents the final step in converting SAH to Hcy [80,81,82]. Hcy is remethylated to methionine through the betaine pathway [83], and then converted into SAM by methionine adenosyltransferase (MAT), thus completing the methionine cycle. Therefore, an increase in NNMT activity accelerates the progression of the methionine cycle, leading to a higher production of Hcy, which, in turn, raises plasma Hcy levels.

#### 2.2.1. Association of Elevated Hcy Levels with Liver Cancer

Although the exact mechanism by which elevated Hcy levels contribute to liver cancer remains incompletely understood, existing evidence suggests a correlation between higher Hcy levels and both liver cancer progression and treatment outcomes. In some adult liver cancer patients with severe liver dysfunction, plasma Hcy levels are often significantly elevated. A study by Stender et al. [84] indicated that patients with an SAH hydrolase deficiency exhibit increased serum transaminase levels, along with significantly elevated levels of serum SAH, SAM, and Hcy. A further study highlighted the pivotal role of Hcy in the methionine cycle, demonstrating its strong correlation with the progression and metastasis of HCC. Elevated levels of Hcy were found to stimulate the proliferation of cancer cells [85]. Furthermore, Zhang et al. [86] hypothesized that the antioxidant transcription factor Nrf2 might be involved in the regulation of Hcy-mediated glutathione (GSH) production in HepG2 human HCC cells. The study utilized the MTT assay to assess the cytotoxicity of Hcy, while Western blotting and immunofluorescence staining was applied to investigate how Hcy affects Nrf2 expression. The results revealed that Nrf2 is involved in mediating the upregulation of GSH levels and enhancing the protective mechanisms in Hcy-induced HepG2 cells. Therefore, elevated Hcy levels are considered an independent factor influencing liver cancer development, and Hcy levels may also serve as a marker for monitoring the progression of liver cancer, offering valuable clinical insights for disease evaluation and prognosis prediction.

#### 2.2.2. Possible Mechanisms Linking Methionine Cycle to Liver Cancer

The disruption of Hcy metabolism may increase susceptibility to diseases like HCC [87]. A substantial body of research has repeatedly demonstrated a significant association between increased Hcy levels and the occurrence of cancer [88], including liver cancer. First, cancer patients typically have elevated plasma Hcy levels. Second, genetic variations in enzymes that participate in Hcy detoxification, including transsulfuration and remethylation, are closely associated with various types of cancer. Third, folate, indispensable for cellular proliferation, is inversely associated with Hcy levels. In the fourth place, Hcy is viewed as a potential indicator for the presence of various cancers. Consequently, the elevation of Hcy levels due to the NNMT-catalyzed methylation of NAM may constitute a pivotal mechanism through which NNMT influences the onset and advancement of hepatocellular carcinoma, highlighting that defects in Hcy metabolism could contribute to cancer development.

In an extensive genome-wide association analysis, Souto et al. [80] discovered that NNMT is a crucial genetic factor influencing the levels of Hcy in plasma. Malaguarnera et al. [89] found that, as chronic hepatitis C (HCV) progresses to cirrhosis and, ultimately, to HCC, serum folate levels gradually decreased, while plasma Hcy levels steadily increased. This phenomenon suggests that both folate and Hcy play crucial roles in disease progression and liver carcinogenesis. The reduction in folate impairs the remethylation of Hcy, leading to decreased SAM concentrations, while the plasma levels of Hcy and SAH rise. These changes collectively result in the hypomethylation of DNA and proteins, particularly histones, thereby influencing gene expression and DNA stability. Under conditions of low or deficient folate levels, plasma Hcy concentrations typically increase. Hcy, an important intermediate in the metabolism of methionine and cysteine, is elevated in a condition known as hyperhomocysteinemia (HHcy) [90]. HHcy may arise from liver disorders or impaired liver function, disrupting intracellular lipid metabolism. Moreover, cytochrome P450 (CYP) arachidonic acid epoxygenase enzymes, found in human cancers, contribute to cancer metastasis. Zhang et al. [91] investigated the interplay between CYP450 metabolism and Hcy in HCC. Their study of 42 HCC samples revealed significantly increased levels of 4-epoxyeicosatrienoic acid (EET) isomers, and the CYP2J2 enzyme responsible for their synthesis and intracellular Hcy were all significantly higher compared to corresponding non-tumor tissues. The decreased DNA methylation induced by Hcy, the essential factors for CYP2J2 expression upregulation, and EET metabolic pathway activation were the ERK1/2 signaling pathway and the SP1/AP1 binding motifs’ activation of the CYP2J2 promoter. Increased Hcy levels promoted enhanced tumor cell characteristics, a phenomenon that was reversed in vitro by silencing CYP2J2. Furthermore, in a mouse model fed 2% (*w*/*w*) L-methionine, with or without folate deficiency, HHcy significantly promoted tumor growth and volume, and an increased CYP2J2 expression, along with DNA demethylation patterns. Knockdown of CYP2J2 using short hairpin RNA (shRNA) markedly weakened these effects. Therefore, HHcy promotes liver cancer development by affecting the DNA demethylation of CYP2J2 and its interaction with the ERK1/2 signaling pathway, thus driving the CYP450-EET metabolic pathway, potentially contributing to the development of hepatic malignancies.

## 3. NNMT-Mediated Regulation of Proliferation, Invasion, and Drug Resistance in Liver Cancer Cells

**Figure 5:**
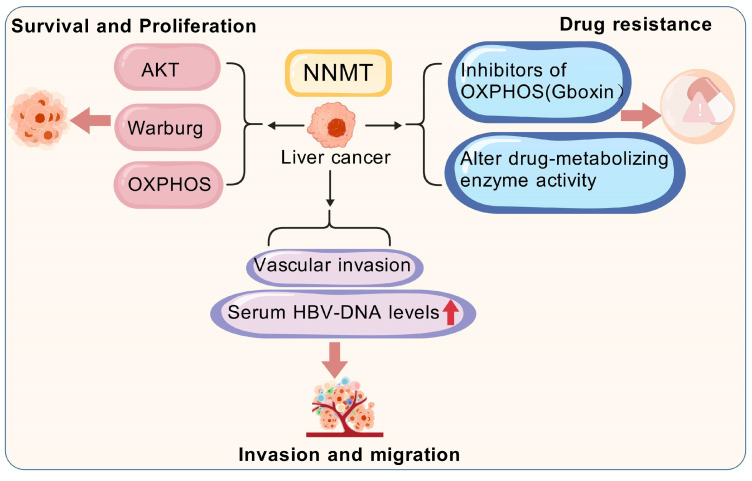
High expression of NNMT and malignant biological behavior of liver cancer cells. NNMT, Nicotinamide n-methyltransferase; AKT, Ak strain Transforming; Warburg, Warburg Effect; OXPHOS, Oxidative Phosphorylation.

NNMT catalyzes the methylation of nicotinamide, participating in energy metabolism and one-carbon metabolic pathways, and demonstrating various biological functions. Within cellular signaling and metabolic regulation networks, NNMT plays a critical role. Specifically, these functions may directly or indirectly regulate a range of malignant behaviors in liver cancer, such as cell survival, proliferation, metastasis, invasion, and drug resistance (Figure 5) [22]. It is noteworthy that, while some studies suggest NNMT is expressed at low levels in liver cancer, others report its high expression. However, the overall trend emerging from recent research points to an increased expression of NNMT in liver cancer.

### 3.1. NNMT Promotes the Survival and Proliferation of Liver Cancer Cells

The NNMT-mediated activation of Akt has a significant effect on the survival and proliferation of liver cancer cells. The promoter region of NNMT contains multiple STAT3 binding sites and a single HNF-1β transcription factor binding site (TFBS), pointing to the possible mechanisms for NNMT expression enhancement in cancer. The continuous activation of STAT3 in different cancers supports tumor growth, progression, and proliferation by regulating multiple signaling pathways, including Akt [92]. For instance, members of the RAC serine/threonine protein kinase (AKT) family, particularly the AKT1 isoform is irregularly expressed in HCC cells and are closely related to biological activities such as survival, proliferation, metabolism, and tumorigenesis [93]. Liang et al. [94] showed that NNMT expression was markedly decreased in HCC tissues relative to adjacent normal tissues. However, in HCC tissues, elevated NNMT expression was closely related to tumor staging, with higher levels observed in recurrent tumors compared to non-recurrent ones. Furthermore, reducing the Warburg effect implies that heightened levels of NNMT could potentially bolster the resilience of cancer cells when subjected to chemotherapeutic treatments. Wu et al. [95] found that the expression levels of NNMT and DNA methyltransferase 1 (DNMT1) are critical in regulating HCC sensitivity to OXPHOS inhibitors. Elevated NNMT expression significantly reduced the sensitivity of these cells to the OXPHOS inhibitor Gboxin. This discovery highlights the pivotal function of NNMT in curbing OXPHOS, a process that liver cancer cells predominantly eschew in favor of glycolysis to fuel their proliferation and sustain their existence. Consequently, an upregulation of NNMT is likely instrumental in bolstering the viability and expansion of these cancerous liver cells.

### 3.2. NNMT Enhances the Invasive and Metastatic Capabilities of Liver Cancer Cells

Previous research has indicated that NNMT acts as an oncogenic in liver cancer, contributing to the invasion and metastasis. Lu et al. [96] identified elevated levels of NNMT in iCCA tissues through Western blotting and immunohistochemistry (IHC). They found that NNMT levels in human iCCA tissues were markedly higher than nearby normal tissues. This finding further confirmed that increased NNMT expression boosts the invasion and metastatic potential of iCCA cells, both in vitro and in vivo. However, the expression pattern and biological role of NNMT in iCCA remain incompletely understood. The potential mechanisms underlying the crosstalk between hepatic stellate cells (HSCs) and liver cancer cells in driving HCC progression are still unclear. Li et al. [97] experimentally demonstrated that activated HSCs boost HCC’s invasive and migratory capabilities by increasing NNMT levels. They co-cultured the human liver cancer cell line SMMC-7721 with conditioned media (CM) from active or dormant HSCs, observing that the active HSC media notably enhanced HCC invasion and metastasis. Furthermore, Li et al. [90] reported that activated HSCs induce the upregulation of NNMT. The contribution of NNMT in modulating various metabolic pathways in liver cancer cells is well-understood. In HCC tissues, elevated NNMT levels are strongly linked to vascular invasion, high serum HBV-DNA, and metastatic spread. A functional analysis revealed that NNMT promotes HCC cell invasion and metastasis by altering histone H3 lysine 27 trimethylation (H3K27me3) patterns and upregulating the cluster of differentiation 44 (CD44) expression. Mu et al. [98] explored the impact of NNMT on the malignant biological behaviors of the human liver cancer cell line SMMC7721. They conducted various experiments, including MTT proliferation assays, colony formation assays, adhesion assays, and invasion assays, using SMMC7721 cells transfected with NNMT (S/NNMT), control SMMC7721 cells (S), and cells transfected with an empty vector (S/P). The MTT assay results showed no significant difference in cell proliferation or colony formation rates between the S/NNMT group and the S or S/P groups (*p* > 0.05). However, the S/NNMT group exhibited a significantly higher heterotypic adhesion ability than the S and S/P groups (*p* < 0.001). Transwell invasion assays showed that the S/NNMT group had a significantly higher optical density (OD) value compared to the other two groups. These results confirmed that NNMT alters the malignant traits of liver cancer cells and enhances their ability to invade and metastasize. Moreover, studies suggest that NNMT is a potential therapeutic target for the statin-induced inhibition of liver cancer cell metastasis. By promoting the Warburg effect, SIRT1 causes cancer cells to prefer glycolysis over oxidative phosphorylation for energy, even when oxygen is present—which also contributes significantly to tumor growth and invasiveness through lactate production [99]. Therefore, NNMT may enhance the Warburg effect, further supporting the invasion and metastasis of liver cancer cells. Gaining a deeper understanding of the metabolic mechanisms of NNMT might reveal new therapeutic targets for treating liver cancer.

### 3.3. NNMT Mediates Drug Resistance in Liver Cancer Cells

Liver cancer cells exhibit significant resistance to a wide range of antitumor drugs, posing a significant challenge in treating liver cancer. This resistance may arise from multiple complex mechanisms, including but not limited to alterations in drug metabolism pathways, shifts in drug targets, and the upregulation of drug transporters. NNMT expression and activity may influence liver cancer cell drug resistance by modulating the activity of drug-metabolizing enzymes. Wu et al. [95] also revealed that NNMT could affect mitochondrial function by regulating NAD^+^ biosynthesis, thereby altering tumor cell sensitivity to mitochondrial oxidative phosphorylation inhibitors. Specifically, the cooperative action of NNMT and DNMT1 can lead to resistance in cancer cells to oxidative phosphorylation inhibitors, such as Gboxin. Wu et al. [95] found that NNMT affects the sensitivity of hepatocellular carcinoma cells to OXPHOS inhibitors by regulating ^NAD+^ metabolism, revealing a direct link between NNMT and mitochondrial function for the first time. However, the study was based only on in vitro cell models and did not validate its conclusions in clinical samples or animal models, and did not explore the synergistic effects of NNMT with other metabolic pathways. The study indicated that, in tumor samples from patients with recurrence, the NNMT expression was elevated while the DNMT1 expression was reduced, resulting in tumor cell resistance to oxidative phosphorylation inhibitors, thus rendering these treatments ineffective and promoting tumor relapse. More specifically, NNMT lowers the methylation potential of cancer cells by depleting SAM, which alters their epigenetic status. This change may trigger modifications in the activity of drug-metabolizing enzymes, ultimately impacting liver cancer cell resistance to treatment [22]. Overall, the research into NNMT’s contribution to drug resistance in liver cancer cells presents novel viewpoints and methods for treating liver cancer. Although many challenges remain, with ongoing research, NNMT may be established as a novel therapeutic target for liver cancer, bringing unprecedented hope to patients.

## 4. Roles of NNMT and Liver Cancer Diagnosis, Treatment, and Prognosis

### 4.1. NNMT in Liver Cancer Diagnosis: A Potential Biomarker

Given the expression profile of NNMT in liver cancer tissues and serum, researchers have explored its potential application in the clinical diagnosis of HCC, suggesting that NNMT may serve as a novel tumor biomarker. Kim et al. [9] conducted an in-depth study on frozen tumor samples and nearby non-cancerous tissues from 120 primary HCC patients. They utilized the real-time reverse transcription PCR (RT-PCR) to measure the expression levels of NNMT and internal control genes, aiming to investigate the correlation between NNMT mRNA levels, clinical pathological parameters, and clinical outcomes. The results revealed that NNMT mRNA levels were significantly lower in HCC tissues compared to adjacent non-cancerous tissues (*p* < 0.0001), and NNMT expression was significantly associated with the tumor stage (*p* = 0.010). Further analysis showed that patients with increased levels of NNMT mRNA showed a tendency for shorter overall survival (OS) (*p* = 0.053) and a marked reduction in disease-free survival (DFS) (*p* = 0.016). These findings suggest that, in HCC cases, NNMT expression is closely linked to tumor stage and DFS duration. Given NNMT’s broad substrate specificity, its expression level may impact both the effectiveness and side effects of chemotherapy. Therefore, NNMT shows promise as a potential biomarker for diagnosing liver cancer and holds significant value in clinical practice.

### 4.2. NNMT in Liver Cancer Treatment: A Promising Therapeutic Target

#### 4.2.1. Pharmacological and Molecular Interventions Targeting NNMT

The role of NNMT in HCC treatment is gaining increasing attention due to its involvement in several critical aspects of tumor biology. One of the most notable areas of interest is NNMT’s influence on ferroptosis in liver cancer cells. Recent studies have demonstrated that NNMT regulates intracellular reactive oxygen species (ROS) levels, which, in turn, modulate the ferroptosis process in liver cancer cells, thereby increasing tumor cell death. Characterized by iron-dependent lipid peroxidation, ferroptosis is a programmed cell death process triggered by excessive ROS production, ultimately leading to cell membrane rupture and cellular demise [100]. Multiple signaling pathways and biological processes are known to contribute to ferroptosis, and NNMT-mediated ROS accumulation has been shown to induce ferroptosis in liver cancer cells, enhancing cell death and modulating the expression of MNAM. This process further influences ROS and lipid peroxidation intensity, accelerating disease resolution. Recent findings suggest that NNMT may influence key ferroptosis pathways, acting as a potential therapeutic target for inhibiting liver cancer cell proliferation and tumor progression [101]. In addition to its effects on cell death, NNMT also plays a significant role in the invasion and metastasis of HCC. Studies have indicated that NNMT promotes the expression of molecules like CD44, which enhances HCC cell invasion and metastasis [97]. It is well-established that NNMT regulates multiple metabolic pathways within liver cancer cells. A high NNMT expression in HCC is significantly linked to vascular invasion, elevated HBV-DNA, and metastatic spread. Studies indicate that NNMT modifies H3K27 methylation, which results in CD44 upregulation and enhances tumor cell invasion and migration. NNMT mediates N6-methyladenosine modifications on CD44 mRNA, promoting the formation of CD44v3 splice variants. Additionally, 1-methyl-nicotinamide, a product of NNMT, helps stabilize the CD44 protein by inhibiting its degradation through ubiquitination. Thus, by modulating the expression and activity of NNMT, it is possible to influence the metastatic potential of liver cancer, providing new insights and strategies for HCC prevention and treatment. Although direct evidence linking NNMT as a therapeutic target for HCC remains lacking, its involvement in tumor initiation and progression, particularly in relation to invasion and metastasis, offers promising new avenues for therapeutic intervention in liver cancer management.

The potential therapeutic approaches targeting NNMT primarily focus on RNA interference (RNAi) drugs and NNMT-inhibiting small molecules [19]. On one hand, RNAi technology can be applied to regulate NNMT expression by designing short hairpin RNAs (shRNAs) or specific siRNAs to selectively suppress NNMT expression. For instance, downregulating NNMT expression through RNAi has been shown to reduce the number of immunosuppressive cells in the TME, potentially boosting the body’s anti-tumor immune response [102]. This approach not only provides a powerful tool for studying the role of NNMT in disease progression but also opens new avenues for developing NNMT-targeted therapeutic strategies. On the other hand, NNMT-targeting small-molecule inhibitors are classified into allosteric inhibitors, dual-substrate competitive inhibitors, SAM competitive inhibitors, and NAM competitive inhibitors (as shown in Table 1). Specifically, NAM-competitive inhibitors vie with NAM for the active site on NNMT. MNAM, a natural NNMT inhibitor, is synthesized in NNMT-mediated reactions [103]. Swaminathan et al. [104] used X-ray crystallography to analyze the ternary complex formed between NNMT and MNAM, revealing how MNAM binds to NNMT’s active site and prevents NAM from binding, thereby inhibiting NNMT’s enzymatic activity. In summary, both RNAi-based therapies and small-molecule inhibitors targeting NNMT have become hot topics in cancer treatment research. Although clinical evidence regarding NNMT’s impact on liver cancer is still limited, its therapeutic potential in this disease should not be disregarded.

If an increased NNMT expression contributes to cancer development, NNMT inhibitors (NNMTis) could potentially suppress the proliferation, migration, and invasion of cancer cells. A recent study [114] explored the effects of NNMTis in an ovarian cancer intraperitoneal metastasis model. While these inhibitors are primarily employed in managing diet-induced obesity connected to heart failure, the study found that NNMTis significantly reduced tumor burden and cell proliferation, along with an enhancement in the trimethylation of histone H3K27. Eckert et al. [29] further emphasized that NNMT promotes cancer progression by regulating the methyl donor balance within tumor cells. Therefore, NNMTis may offer therapeutic advantages by modulating the epigenome of cancer cells, presenting a promising avenue for liver cancer treatment. Neelakantan et al. [12] discovered that systemic quinoline-derived NNMT inhibitors markedly decreased body weight and adiposity in an obesity mouse model induced by diet, and also optimized plasma lipid levels. This implies that integrating NNMT inhibitors with dietary measures, like NAD^+^ precursor supplements, may boost treatment outcomes. This combination approach may also allow for a dose reduction in the NNMT inhibitors, thereby potentially lowering the risk of side effects. Similarly, Kantt et al. [117] noted that JBSNF-00008 treatment in obese mice on a high-fat diet with a nicotinamide-derived NNMT inhibitor experienced weight reduction, enhanced insulin sensitivity, and restored glucose tolerance. Combining these NNMT inhibitors with chemotherapy agents might further enhance the effectiveness of traditional chemotherapy. Several studies have demonstrated that the inhibition of NNMT can enhance the sensitivity of cancer cells to chemotherapy [13,123,124]. By inhibiting NNMT, it may be possible to modulate tumor cell metabolism, making them more responsive to chemotherapy. Additionally, NNMT inhibition is vital for metabolic regulation [125]. Specifically, knockdown of NNMT expression increases energy expenditure in adipocytes [34] and decreases glucose output in hepatocytes [57]. NNMT knockout in animal models has markedly enhanced fasting blood glucose levels, glucose tolerance, and insulin sensitivity, and, also, triglycerides, and decreased lipid deposition [34,57,126]. These findings indicate that silencing NNMT could offer a novel strategy for treating metabolic disorders.

Overall, NNMT could be a valuable target for creating oligonucleotide-based cancer therapies. However, the effective implementation of oligonucleotide treatment still faces several significant technical challenges [127]. Challenges encompass accurate oligonucleotide targeting to specific tissues or organs and regulating interactions with their molecular targets [128,129], minimizing the toxicity associated with oligonucleotide sequences and their chemical properties, as well as preventing the saturation of natural RNA processing pathways [130]. While therapies based on oligonucleotides targeting NNMT have shown encouraging therapeutic outcomes in preclinical and animal studies, they have yet to progress to clinical trials. In contrast, small-molecule NNMT inhibitors have shown substantial improvements in selectivity and efficacy. NNMT’s extensive clinical implications in cancer and metabolic diseases have prompted the pursuit of more potent NNMT inhibitors. While still in the initial stages of preclinical trials, these compounds highlight the ongoing endeavors to address the difficulties related to oligonucleotide therapeutics. Consequently, the development of oligonucleotide drugs against NNMT is an auspicious approach for upcoming liver cancer therapies.

#### 4.2.2. Exercise Therapy Targeting NNMT: A Non-Pharmacological Approach

Our study suggests that exercise can be used as a non-pharmacological strategy to modulate NNMT activity [131,132]. In rodent models, aerobic exercise decreases hepatic NNMT expression and improves insulin sensitivity through AMPK-SIRT1 signaling [133]. Clinical observations further suggest that patients with HCC who regularly exercise at a moderate intensity have lower serum NNMT levels and longer progression-free survival compared with sedentary patients [134]. Mechanistically, exercise enhances anti-tumor immunity by reducing immunosuppressive cells in the TME [135], which may have synergistic effects with NNMT-targeted drugs. Despite these benefits, challenges such as optimizing exercise regimens (e.g., intensity and duration) and ensuring patient compliance require further investigation.

### 4.3. NNMT in Liver Cancer Prognosis: A Prognostic Indicator

The abnormal overexpression of NNMT is closely associated with adverse prognostic characteristics in HCC patients. These characteristics include vascular invasion, cirrhosis, elevated serum HBV levels, and distant metastasis. Kim et al. [9] conducted a study stratifying HCC samples based on NNMT expression levels. The multivariate analysis results indicated that NNMT expression (*p* = 0.0096) and advanced tumor stage (*p* = 0.0017) were significant predictors of DFS. Additionally, Dang and colleagues [136] conducted a meta-analysis to assess the prognostic significance of NNMT expression in HCC and other tumors. Their findings showed that a high NNMT expression, compared to a low expression, was significantly correlated with shorter OS, DFS, and clinical pathological parameters (CP) in cancer patients. These parameters encompassed less differentiated tumors, earlier distant metastasis, earlier lymph node metastasis, and later clinical staging. This suggests that a high NNMT expression could be a marker of poor prognosis in HCC patients. Moreover, NNMT expression could serve as a prognostic marker for various cancers, particularly liver cancer, and potentially as a therapeutic target. Therefore, NNMT expression levels might be a key prognostic indicator for patients with HCC. In HCC, a further investigation of the molecular interactions between NNMT and various pathogenic factors will provide valuable insights and support the development of effective treatment strategies.

A detailed overview of pivotal studies was compiled in Table 2 to comprehensively summarize the current evidence for NNMT in the diagnosis, treatment, and prognosis of HCC.

## 5. Future Research Directions and Recommendations

This study delves into the central role of NNMT in driving liver cancer progression and proposes it as a promising therapeutic target for both the prevention and treatment of liver cancer. While numerous positive findings have emerged, there remain many unanswered questions regarding the precise mechanisms through which NNMT influences liver cancer and how effective clinical drugs targeting NNMT can be designed.

Currently, the understanding of how NNMT influences anaerobic energy metabolism remains limited. The inhibition of NNMT has been reported to affect the bioenergetics of endothelial cells [137]. A notable characteristic of tumor cell metabolism is the abnormal increase in anaerobic glycolysis, which leads to a significant accumulation of energy derived from glucose, regardless of oxygen levels. These metabolic alterations facilitate rapid cell proliferation and tumor growth, further confirming the process of the Warburg effect [138]. Many studies stress NNMT’s key role in curbing aerobic glucose and lipid metabolism; yet, its function in anaerobic pathways remains undefined. Our previous research [132] indicated that NNMT levels are elevated in the extensor digitorum longus, which mainly depends on anaerobic metabolism, when compared to the soleus muscle, which mainly utilizes oxidative metabolism. Moreover, using MNAM to suppress NNMT has led to lower performance in anaerobic endurance experiments in rats [139]. These findings suggest that NNMT plays a significant role in modulating anaerobic metabolism, potentially influencing exercise performance, particularly in activities that demand high-intensity, anaerobic energy production.

A critical gap lies in elucidating the functional heterogeneity of NNMT across tumor types. For instance, systematic comparisons between HCC and NNMT-high malignancies (e.g., breast and ovarian cancers) could delineate conserved pathways (e.g., NAD^+^ salvage mechanisms) versus liver-specific vulnerabilities, such as the GNMT–NNMT regulatory axis in methionine cycle homeostasis. While NNMT inhibition reprograms immunosuppressive niches in ovarian cancer [102], its immunomodulatory effects on HCC-associated macrophages or stromal compartments remain unexplored. Furthermore, tissue-selective delivery strategies (e.g., hepatocyte-targeted RNAi versus pan-cancer NNMT inhibitors) may enhance therapeutic precision by balancing efficacy and toxicity profiles. Collectively, these investigations will clarify whether NNMT operates as a universal metabolic linchpin or a context-dependent orchestrator of oncogenesis.

Further research is essential for evaluating the potential applicability of NNMT as a therapeutic target for liver cancer. Challenges have arisen in developing RNAi-based therapies and small-molecule NNMT inhibitors. In RNAi drug development, these challenges include issues related to safety, efficacy, and delivery systems. To overcome these hurdles, researchers have explored various delivery strategies, including viral and non-viral vectors, as well as chemical modifications, cationic liposomes, polymers, nanoparticles, and bio-conjugation methods for siRNA to enhance stability and intracellular delivery [140]. Several RNAi-based drugs, such as the aptamer pegaptanib targeting specific proteins, microRNA inhibitors like givosiran and patisiran, and antisense oligonucleotides targeting RNA, such as golodirsen and inotersen, have been approved by the U.S. Food and Drug Administration (FDA) for clinical use [141]. While RNAi therapeutics have seen considerable advancement clinically, enhancements in pharmacokinetics, pharmacodynamics, and toxicity mitigation are still needed [142]. For small-molecule inhibitors of NNMT, the challenge lies in identifying inhibitors that are both highly effective and selective, with robust activity within living organisms [143]. Currently, small-molecule NNMTi are widely used to study the pharmacological effects of NNMT, but their effectiveness is questionable due to problems with inadequate selectivity and poor metabolic stability [144].

Overall, compared to a recently published NNMT review [145,146], this article systematically sorted out the mechanism of NNMT regulating immune escape in liver cancer through metabolic reprogramming for the first time, and proposed its potential as a target for combination therapy. In addition, we summarize in detail the barriers to the clinical translation of small-molecule inhibitors and RNAi therapeutics for NNMT, providing a clear direction for future research.

## 6. Conclusions

Despite evidence indicating NNMT expression in liver cancer and its role in tumor proliferation and metastasis, the precise mechanisms underlying its influence on cancer progression remain poorly understood. Therapies targeting NNMT, including small-molecule inhibitors and RNAi approaches, have shown promising potential in inhibiting key oncogenic processes such as cell survival, proliferation, invasion, metastasis, and drug resistance. However, several major obstacles hinder the clinical translation of these findings. The lack of reliable biomarkers with which to identify patients most likely to respond to NNMT-targeted therapies represents a significant challenge. Additionally, the pharmacokinetics and tumor penetration of current NNMT inhibitors need optimization, and their long-term safety and efficacy in humans remain to be determined. Therefore, further investigation is crucial for elucidating NNMT’s role in liver cancer and advancing the development of targeted therapeutic strategies. Understanding how NNMT regulates the NAD^+^ biosynthesis pathway and the methionine cycle could provide new insights into liver cancer pathogenesis and treatment.

## Figures and Tables

**Table 1:** Small-molecule inhibitors of nicotinamide N-methyltransferase (NNMT).

NNMT Inhibitor Type	Compound	References
Allosteric inhibitors	Macrocyclic peptides	[105]
Dual-substrate-competitive inhibitors	MvH45, AK-12, MS2756, MS2734, GYZ-78, NS1, LL320, Yuanhuadine *, CC-410,GYZ-319, Para-cyano compound17u	[103,106,107,108,109,110,111,112,113,114]
NAM-competitive inhibitors	MNAM, 5-amino-1MQ (NNMTi), jBSNF-000088, JBSNF-000265, JBSNF-000028, RS004,4-chloro-3-ethynylpyridine	[12,34,57,107,115,116,117,118,119,120]
SAM-competitive inhibitors	SAH, sinefungin *	[121,122]

* A natural inhibitor.

**Table 2:** Role of NNMT in the diagnosis, treatment, and prognosis of liver cancer.

Models and References	Sample Size	Key Findings	Limitations
Tumor tissues of 120 HCC patients [9]	120	The mRNA level of NNMT in HCC tissues was significantly lower than that in adjacent tissues, but the serum level was negatively correlated with prognosis.	The mechanism of tissue and serum expression differences was not explained, and dynamic monitoring data were lacking.
SMMC-7721 cells in mouse metastasis model [97]	92	Activation of HSCs promotes HCC invasion by upregulating NNMT.	Correlation in clinical samples was not verified.
MCC-13 and MCC-26 cells [123]	11	NNMT may be a promising target for Merkel cell carcinoma biomarkers and targeted anti-cancer therapies.	Further experiments are needed to understand the specific mechanisms by which NNMT is involved in the development of Merkel cell carcinoma.
Human OS cell lines U-2 OS and Saos-2 [124]	20	NNMT promotes tumor cell proliferation, migration, and chemoresistance of osteosarcoma (OS) cells.	Due to the rarity of osteosarcoma (OS), immunohistochemical analysis involves a limited number of patients.
Human malignant melanoma cell line A375 [13]	N/A	NNMT is somehow involved in the mechanisms that promote chemoresistance of melanoma cells.	Further analysis is needed to gain a deeper understanding of the mechanisms by which this enzyme is involved in melanoma tumorigenesis
NNMT expression knockout [126]	N/A	Knockdown of NNMT expression reduced lipid accumulation and triglyceride content in 3T3-L1 cells.	Long-term side effects were not assessed, and differences in human metabolism were not considered.

N/A, not applicable.

## Data Availability

No new data were created or analyzed in this study.
